# Factors associated with disease control failure in acromegaly patients treated with pegvisomant: an ACROSTUDY analysis

**DOI:** 10.1530/EC-23-0247

**Published:** 2024-01-29

**Authors:** Antonella Giampietro, Sabrina Chiloiro, Claudio Urbani, Rosario Pivonello, Martin Ove Carlsson, Francesca Dassie, Nunzia Prencipe, Marta Ragonese, Roy Gomez, Simona Granato, Salvatore Cannavò, Silvia Grottoli, Pietro Maffei, Annamaria Colao, Fausto Bogazzi, Antonio Bianchi

**Affiliations:** 1 Pituitary Unit, Department of Endocrinology, Fondazione A Gemelli, IRCCS, Università Cattolica del Sacro Cuore, Rome, Italy; 2 Endocrinology II Unit, Department of Medicine, Azienda Ospedaliero Universitaria Pisana, Pisa, Italy; 3 Dipartimento Di Medicina Clinica E Chirurgia, Sezione Di Endocrinologia, Università Federico II di Napoli, Naples, Italy; 4 Global Medical Affairs, Pfizer Rare Disease, Brussels, Belgium; 5 Department of Medicine - DIMED, University of Padua, Padua, Italy; 6 Division of Endocrinology, Diabetology and Metabolism, Department of Medical Science, University of Turin, Turin, Italy; 7 Unit of Endocrinology, Department of Human Pathology, University of Messina, Messina, Italy; 8 Medical Department, Pfizer Italia, Rome, Italy; 9 Department of Clinical and Experimental Medicine, University of Pisa, Pisa, Italy

**Keywords:** acromegaly, longitudinal studies, pegvisomant, real-world analysis

## Abstract

**Purpose:**

The aim of this study was to examine the probability of achieving acromegaly disease control according to several patient-, disease- and treatment-related factors longitudinally.

**Methods:**

We analyzed data from ACROSTUDY, an open-label, noninterventional, post-marketing safety surveillance study conducted in 15 countries. A total of 1546 patients with acromegaly and treated with pegvisomant, with available information on baseline IGF-1 level, were included. Factors influencing IGF-1 control were assessed up to 10 years of follow-up by mixed-effects logistic regression models, taking into account changing values of covariates at baseline and at yearly visits. Twenty-eight anthropometric, clinical and treatment-related covariates were examined through univariate and multivariate analyses. We tested whether the probability of non-control was different than 0.50 (50%) by computing effect sizes (ES) and the corresponding 95% CI.

**Results:**

Univariate analysis showed that age <40 years, normal or overweight, baseline IGF-1 <300 µg/L or ranged between 300 and 500 µg/L, and all pegvisomant dose <20 mg/day were associated with a lower probability of acromegaly uncontrol. Consistently, in multivariate analyses, the probability of uncontrolled acromegaly was influenced by baseline IGF-1 value: patients with IGF-1 <300 µg/L had the lowest risk of un-controlled acromegaly (ES = 0.29, 95% CI: 0.23–0.36). The probability of acromegaly uncontrol was also lower for values 300–500 µg/L (ES = 0.37, 95% CI: 0.32–0.43), while it was higher for baseline IGF-1 values ≥700 µg/L (ES = 0.58, 95% CI: 0.53–0.64).

**Conclusion:**

Baseline IGF-l levels were a good predictor factor for long-term acromegaly control. On the contrary, our data did not support a role of age, sex, BMI and pegvisomant dose as predictors of long-term control of acromegaly.

**Significance statement:**

Among factors that could influence and predict the efficacy of pegvisomant therapy in controlling acromegaly, a central role of baseline IGF-1 values on the probability of achieving a biochemical control of acromegaly during the treatment with pegvisomant was identified, in a real-life setting.

## Introduction

Acromegaly is a chronic, slowly progressive disease characterized by somatic disfigurement, particularly of the extremities, and systemic manifestations (1). In most cases, acromegaly occurs as a consequence of a growth hormone (GH)-secreting pituitary tumor that increases GH and insulin-like growth factor 1 (IGF-1) secretion (2). The primary aim of the treatment – through neurosurgery, radiotherapy or pharmacological therapy – is to achieve disease control by normalizing GH and IGF-1 concentrations (2, 3).

Achieving disease control in subjects with acromegaly is essential to lengthen patient survival and decrease mortality (4, 5, 6, 7). Furthermore, disease control is a key factor to reduce the risk of systemic comorbidities of acromegaly, improve the quality of life of patients, as well as reduce the economic burden of disease in terms of both direct and indirect costs (8, 9). Notwithstanding major improvements in disease management over the last decades, national acromegaly registries and real-world investigations reported that 30–40% of patients still fail to achieve disease control (4, 10, 11, 12, 13, 14). More recently, promising results were reported by European and American centers, where multimodality therapy achieved a biochemical control in over 70% of patients with acromegaly (15, 16).

Information on factors predicting the response to medical therapies in terms of disease control is still scanty. A few analyses suggested that pretreatment IGF-1 levels, sex, body mass index (BMI) and previous radiotherapy may play a role in the efficacy/effectiveness of pharmacologic treatments to achieve disease control (17, 18, 19, 20, 21). However, results were inconsistent between studies, and this topic is open to discussion.

Thus, the aim of this analysis was to examine which factors are associated with the achievement of disease control using data from ACROSTUDY, a large international study of pegvisomant-treated patients with acromegaly.

## Materials and methods

ACROSTUDY is an open-label, noninterventional, post-marketing safety surveillance study of pegvisomant-treated patients with acromegaly. The primary aim of the study is to monitor the long-term safety and effectiveness of pegvisomant in the real-world clinical practice. In this observational study, data were collected as part of the routine clinical care of each patient. All treatment-related aspects and the visit schedule were at the discretion of treating physicians. Detailed information on the methods of ACROSTUDY have been provided elsewhere (22, 23). The study was conducted in compliance with the Declaration of Helsinki and with applicable local laws and requirements. Ethical approval from local Boards or Ethical Committees was obtained for all study centers; the list of ethics committees was previously published (24). All patients provided written informed consent before enrollment in the study.

All patients with acromegaly included in the study were already under treatment or were starting pegvisomant at enrolment in ACROSTUDY. Exclusion criteria were participation in other trials of acromegaly, the requirement of surgery to decompress the tumor for visual field loss, cranial nerve palsies, intracranial hypertension and rhinoliquorrhea.

This investigation was based on the complete analysis set of ACROSTUDY, including data from 15 countries for a total of 2221 enrolled patients. Information on IGF-1 status (i.e. below the lower limit of normal, normal or above the upper limit of normal, based on local laboratories’ data) at baseline was missing for 675 patients. These patients were excluded from the present analysis, leaving a total of 1546 acromegaly patients. Data analysis was restricted to 10 years of study follow-up (at the 10-year visit, *n* = 193 patients had data on IGF-1 status) due to the limited number of available data-points thereafter. In particular, the sample size was 1546, 1220, 1208, 1078, 957, 799, 613, 437, 320, 257 and 193 at years 0, 1, 2, 3, 4, 5, 6, 7, 8, 9 and 10, respectively.

Data were collected through an electronic case report form (eCRF) using a web-based tool, at baseline (using clinical records) and during follow-up visits. The main data collected in ACROSTUDY were sociodemographic features (e.g. age, sex, race); personal patient characteristics (e.g. weight, height, BMI computed as weight in kilograms/height in meters × height in meters), disease-related information (e.g. date of diagnosis and symptoms, physical examination data, pituitary imaging features, dose of pegvisomant), presence of comorbidities (e.g. hypertension, diabetes, cardiovascular conditions, neoplasms, respiratory diseases, osteoarthritis), laboratory tests (e.g. ALT, AST, IGF-1 levels, GH at baseline, HbA1c) and quality of life (22, 23). According to the protocol, IGF1 was recorded at baseline, defined as 6 months before and 1 day after pegvisomant start, after 6 months of pegvisomant treatment and every 6 months thereafter (22, 23) (Supplementary materials). Information on concomitant acromegaly treatments before and during pegvisomant therapy was also collected. For this analysis, medications for acromegaly were classified into four categories (pegvisomant only, pegvisomant plus somatostatin analogs (SSA), pegvisomant plus other non-SSA drugs, pegvisomant plus SSA plus other drugs). The list of ‘other drugs’ is provided in Supplementary materials (see the section on supplementary materials given at the end of this article).

Laboratory tests were carried out at the discretion of the treating physician in ACROSTUDY. They were conducted using commercial assays available at each study center (a total of six different commercial tests were utilized) and the results were interpreted according to their normal reference ranges. IGF-1 data were reported as absolute values as well as normalized to their relative upper limit of age-adjusted normal values. IGF-1 levels at baseline were also harmonized centrally (to µg/L) in order to allow for the inclusion of this information in the multivariate models. IGF-1 control was indeed analyzed longitudinally as a binary outcome, not as a continuous variable. If any one of the assessments of IGF-1 was either greater than the upper limit of normal (ULN) or less than the lower limit of normal (LLN), the patient was defined as ‘IGF-1 uncontrolled’. Otherwise, the patient was defined as ‘IGF-1 controlled’. Disease control at baseline and at each follow-up visit was defined as an IGF-1 value below the age- and sex-specific upper limit of normal (i.e. an IGF-1 index less than 1).

IGF-1 standard deviation scores (SDS) were not derived owing to the fact that different types of assays were used.

### Statistical analysis

IGF-1 control was assessed longitudinally using mixed-effects logistic regression models to account for the correlated structure of repeated measures data and changing values of covariates at baseline and at yearly visits, up to 10 years of follow-up. A total of 28 sociodemographic, anthropometric and clinical covariates were initially considered in a univariate longitudinal analysis. Variables that were significant at the 10% level were included in the multivariate analysis, including time × covariate interaction effects. The final model applied 5% level of significance, testing whether the probability of non-control (i.e. the effect size, ES) was different than 0.50 (50%), and computed 95% CI. Two covariates were thus retained in the final multivariate model, i.e. medications for acromegaly and IGF-1 value at baseline. As ES, the probability of non-control of disease was computed (i.e. an estimated probability <0.50 indicates a favorable effect on acromegaly disease control). Multilevel logistic regression was fit by the GLIMMIX procedure in SAS v.9.4 (SAS Institute Inc., Cary, NC, USA) using maximum likelihood estimation. Estimates of the average logits in the groups and their predictions on the scale of the data were produced with the LSMEANS statement and ILINK option in PROC GLIMMIX. The performance of the multivariate longitudinal mixed-effects logistic regression model was evaluated through receiver operating characteristic (ROC) curves by computing the area under the curve (AUC) (25). Student’s *t*-test with a two-sided 5% significance level was applied to compare groups of patients with a controlled or an uncontrolled disease at the last visit (end of follow-up) for continuous variables. Welch’s *t*-test was applied in the case of unequal variances. Chi-squared test was used for categorical variables.

## Results

Table 1 summarizes the main characteristics of acromegaly patients at baseline and compares patients with controlled or uncontrolled disease at the last visit or at the end of follow-up. A total of 1546 subjects (48% females) were included in the analysis. Their mean age at diagnosis of acromegaly and at the introduction of pegvisomant was 42.1 ± 13.7 years and 49.4 ± 14.3 years, respectively. At baseline, the mean BMI was 29.4 kg/m^2^ (s.d. 5.5), with 30.5% of patients being obese, according to BMI. The mean IGF-1 value at baseline was 513.1 ± 314.6, and 11.6% of patients had controlled disease at baseline. At baseline, out of 1546 patients, 838 (54.2%) were initially prescribed with pegvisomant only and 708 (45.8%) with a combination therapy (34.5% with SSA, 5.5% with other drugs and 5.8% with SSA plus another drug). The mean pegvisomant starting dose was 11.1 ± 7.5 mg/day. Patients with a controlled disease at the last visit/end of follow-up were older at the diagnosis of acromegaly (*P* = 0.03) and at pegvisomant start (*P* = 0.005) than patients with an uncontrolled disease. Furthermore, patients with controlled disease had significantly lower BMI (*P* = 0.005) and IGF-1 value at baseline (*P* < 0.001) compared to subjects with not controlled disease. Concerning baseline treatment characteristics, those with a controlled disease at the last visit/end of follow-up were treated more frequently with pegvisomant as a monotherapy (57.1 vs 50.6 of combined regimen) and at a lower dose (*P* = 0.01) than patients with uncontrolled acromegaly at the last evaluation.

**Table 1:** Baseline characteristics of 1546 acromegaly patients included in the analyses, overall and according to disease control at the last visit/end of follow-up.

Characteristics	Total	Patients not controlled at last visit/end of follow-up	Patients controlled at last visit/end of follow-up	Controlled vs uncontrolled patients
Sex, *n* (%)				
Female	743 (48.1)	327 (47.4)	416 (48.6)	
Male	803 (51.9)	363 (52.6)	440 (51.4)	
*P*				0.6368
Age at acromegaly diagnosis, mean ± s.d.	42.1 ± 13.7	41.2 ± 13.8	42.7 ± 13.6	0.0304
Age at pegvisomant start, mean ± s.d.	49.4 ± 14.3	48.2 ± 14.9	50.3 ± 13.8	0.0050
Body mass index at baseline (kg/m^2^), mean ± s.d.	29.4 ± 5.5	29.8 ± 5.8	28.9 ± 5.1	0.0046
Body mass index (kg/m^2^), *n* (%)				
<25	257 (16.6)	107 (15.5)	150 (17.5)	
25-<30	491 (31.8)	222 (32.2)	269 (31.4)	
30+	471 (30.5)	241 (34.9)	230 (26.9)	
*P*				0.0324
Missing	327 (21.1)	120 (17.4)	207 (24.2)	
IGF-1 value at baseline (µg/L), mean ± s.d.	513.1 ± 314.6	573.2 ± 339.2	464.6 ± 284.3	<0.0001
Medications for acromegaly, *n* (%)				
Peg only	838 (54.2)	349 (50.6)	489 (57.1)	
Peg/other (non-SSA)	85 (5.5)	43 (6.2)	42 (4.9)	
Peg/SSA	533 (34.5)	238 (34.5)	295 (34.5)	
Peg/SSA/other	90 (5.8)	60 (8.7)	30 (3.5)	
*P*				<0.0001
Pegvisomant starting dose (mg), mean ± s.d.	11.1 ± 7.5	11.7 ± 9.0	10.7 ± 5.9	0.0102

Supplementary Table 1 (see the section on supplementary materials given at the end of this article) shows the mean pegvisomant daily dose in two patient subcohorts treated during the whole period of the first 24 months with (i) pegvisomant only and (ii) pegvisomant plus SSA, according to disease control at different time points. In both subcohorts, pegvisomant dose tended to increase from baseline to months 12 and 24, and – at month 24 – the mean daily dose was lowest in the subgroups of patients that had controlled disease at both time points (15.1 mg/day in the subcohort of patients treated with pegvisomant only and 12.1 in those treated with pegvisomant plus SSA). In both subcohorts, after 12 months of treatment, there was no meaningful difference in the pegvisomant dose between controlled vs not controlled disease. The same lack of differences in the pegvisomant dose between controlled vs not controlled disease at 24 months for patients who were controlled at 12 months and between controlled vs not controlled disease at 24 months for patients who were not controlled at 12 months was observed.

Table 2 illustrates the results of univariate analyses on the impact of the selected factors on acromegaly control. Disease control was achieved by 56.5% of patients with available data at year 1, 61.5% at year 3, 65.2% at year 5 and 76.4% at year 10. The effect size of both sexes separately analyzed (females: ES = 0.40, 95% CI: 0.37–0.43; males: ES = 0.42, 95% CI: 0.39–0.45) and of age ≥40 years at the start of pegvisomant (age 40–49, ES = 0.44; age 50–59, ES = 0.37; age ≥60 years, ES = 0.35) was lower 0.50, thus indicating a lower probability of having an uncontrolled disease over time in presence of these characteristics. The probability of failing the control of acromegaly was also lower in normal weight (ES = 0.35, 95% CI: 0.30–0.41) or overweight patients (ES = 0.40, 95% CI: 0.36–0.44) but not in obese (ES = 0.52, 95% CI: 0.48–0.56). The presence of elevated random GH levels did not affect the probability of not controlling the disease, as in patients both without (ES = 0.36, 95% CI: 0.29–0.43) and with (ES = 0.44, 95% CI: 0.41–0.47) elevated random GH, the effect size was in favor to disease control. On the contrary, in respect of baseline IGF-1 levels, a lower probability of not controlling acromegaly was observed only in patients with baseline IGF-1 value <300 µg/L (ES = 0.24, 95% CI: 0.20–0.29) or between 300 and <500 µg/L (ES = 0.36, 95% CI: 0.32–0.40). Similarly, pegvisomant alone (ES = 0.37, 95% CI: 0.35–0.40) and pegvisomant plus SSA (ES = 0.41, 95% CI: 0.38–0.45) had an effect size in favor to disease control. In univariate analyses, such probability was also lower in patients treated with a mean daily dose of pegvisomant <20 mg (ES = 0.32 for <10 mg, ES = 0.33 for 10–15 mg, and ES = 0.42 for 15–20 mg), while it was higher in those treated with ≥20 mg/day (ES = 0.56, 95% CI: 0.52–0.59).

**Table 2:** Univariate findings of control of acromegaly, according to selected factors.

Characteristics	Univariate analyses			
	Effect size^a^	95% CI		*P*
Sex				
Female	0.40	0.37	0.43	<0.0001
Male	0.42	0.39	0.45	<0.0001
Age at pegvisomant start				
<30	0.44	0.37	0.51	0.0848
30–39	0.49	0.45	0.54	0.8053
40–49	0.44	0.40	0.48	0.0060
50–59	0.37	0.33	0.41	<0.0001
60+	0.35	0.31	0.39	<0.0001
Body mass index (kg/m^2^)				
<25	0.35	0.30	0.41	<0.0001
25–30	0.40	0.36	0.44	<0.0001
30+	0.52	0.48	0.56	0.3673
Elevated random GH				
No	0.36	0.29	0.43	0.0001
Yes	0.44	0.41	0.47	<0.0001
IGF-1 value at baseline (µg/L)				
<300	0.24	0.20	0.29	<0.0001
300–500	0.36	0.32	0.40	<0.0001
500–700	0.50	0.45	0.55	0.9205
700+	0.56	0.52	0.61	0.0068
Medications for acromegaly				
Peg only	0.37	0.35	0.40	<0.0001
Peg/other (non-SSA)	0.48	0.42	0.54	0.5292
Peg/SSA	0.41	0.38	0.45	<0.0001
Peg/SSA/other	0.57	0.49	0.64	0.0756
Pegvisomant mean dose during study (mg)				
<10	0.32	0.28	0.36	<0.0001
10–15	0.33	0.30	0.37	<0.0001
15–20	0.42	0.38	0.46	<0.0001
20+	0.56	0.52	0.59	0.0029

^a^Effect size <0.50 indicates a favorable effect on acromegaly control.

**Figure 1:**
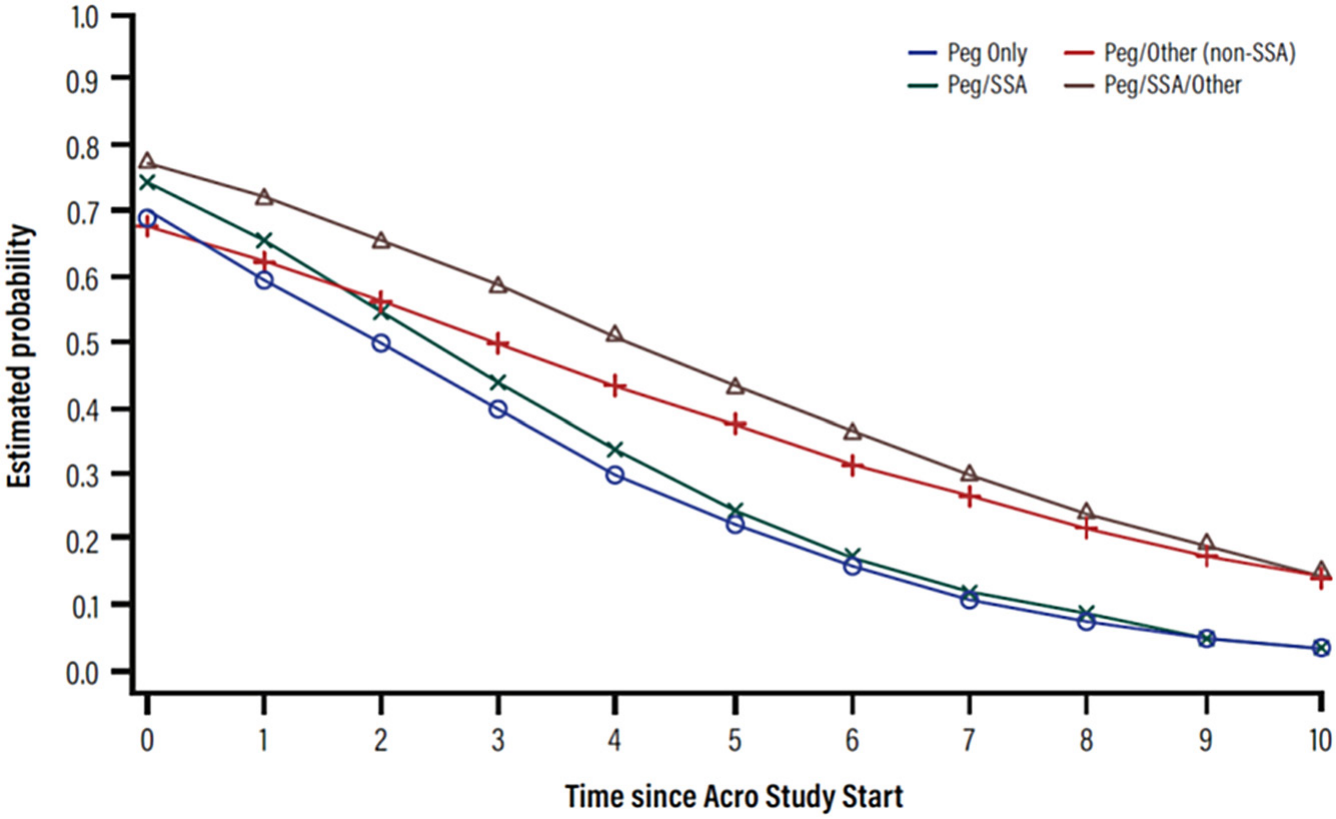
Probability of non-control of IGF-1 over time, according to the use of different medications for acromegaly.

Figure 1 shows the probability of uncontrolled acromegaly over the years, according to the use of different medical therapies for acromegaly, assessed by univariate analyses. The probability of uncontrolled disease was highest throughout the whole follow-up for patients treated with pegvisomant plus SSA and other drugs and lowest for most of the time points in those treated with pegvisomant as a monotherapy.

The results of the multivariate analysis of the factors influencing acromegaly control are given in Table 3. Among all the factors showing an effect through the univariate analysis, only the type of medical treatment at each time point and the baseline IGF-1 values had an impact on disease control after their inclusion in the multivariate modeling. The mean dose of pegvisomant at the last visit/end of follow-up tended to increase with the baseline IGF-1 value, from 12.3 mg/day in patients with IGF-1 level <300 µg/L to 18.7 mg/day in those with IGF-1 ≥700 µg/L at baseline. The probability of un-controlled disease during the first 10 years of ACROSTUDY was lower for patients treated with pegvisomant (ES = 0.35, 95% CI: 0.32–0.39) as well as for those treated with pegvisomant plus SSA (ES = 0.44, 95% CI: 0.39–0.50). In pairwise comparisons between different types of medical treatments for acromegaly, monotherapy with pegvisomant had a significantly lower probability of uncontrolled acromegaly than all combination therapies (all *P*-values <0.01). The probability of disease control was dependent on baseline IGF-1 value: patients with IGF-1 values <300 µg/L had the lowest risk of not controlling acromegaly at the last visit/end of the follow-up (ES = 0.29, 95% CI: 0.23–0.36; in pairwise comparisons, all *P*-values were <0.05 vs other groups of IGF-1 level at baseline). The probability of uncontrolled acromegaly was also lower in those with IGF-1 levels 300–500 µg/L (ES = 0.37, 95% CI: 0.32–0.43), while it was significantly increased in those with IGF-1 values ≥700 µg/L at baseline (ES = 0.58, 95% CI: 0.53–0.64).

**Table 3:** Multivariate analysis of factors associated with acromegaly control during the first 10 years in ACROSTUDY.

		Multivariate analysis				Pairwise comparison
Characteristics	Mean ± s.d. Peg dose at last visit/end of follow-up	Effect size^a^	95% CI		*P*	*P*
Medications for acromegaly						
Peg only	15.3 ± 9.1	0.35	0.32	0.39	<0.0001	Reference
Peg/other (non-SSA)	19.3 ± 12.3	0.47	0.39	0.55	0.4182	0.0074
Peg/SSA	14.3 ± 9.7	0.44	0.39	0.50	0.0378	0.0015
Peg/SSA/Other	22.1 ± 17.4	0.50	0.41	0.59	0.9683	0.0031
Interaction between time and medications for acromegaly		–	–	–	0.0061	
IGF-1 value at baseline (µg/L)						
<300	12.3 ± 8.8	0.29	0.23	0.36	<0.0001	Reference
300–500	14.5 ± 9.1	0.37	0.32	0.43	<0.0001	0.0282
500–700	15.5 ± 10.9	0.53	0.47	0.59	0.3829	<0.0001
700+	18.7 ± 13.4	0.58	0.53	0.64	0.0041	<0.0001
Interaction between time and IGF-1 value at baseline		–	–	–	0.0551	

^a^Effect size <0.50 indicates a favorable effect on acromegaly control.

When a second analysis was run, excluding patients who are IGF-1 controlled at baseline, the results indicated lower, not statistically significant, effect sizes for uncontrolled disease in all treatment groups compared to the full dataset. The ES for Peg only, Peg/other (non-SSA), Peg/SSA and Peg/SSA/other were 0.29. 0.40, 0.37 and 0.42 compared to 0.35, 0.47, 0.44 and 0.50 for the full dataset.

A role of the mean daily dose of pegvisomant on the probability of disease control emerged in preliminary multivariate analyses, with a higher probability of control in patients treated with lower doses (data not shown). However, this effect vanished when IGF-1 values at baseline were included (i.e. adjusted for) in the multivariate models.

Supplementary Table 2 reports the mean daily dose of pegvisomant at different treatment times, separately for patients with controlled and uncontrolled acromegaly. Pegvisomant dose was higher in uncontrolled than in controlled patients after 3 years (18.1 vs 15.0 mg/day, respectively, *P* < 0.001), 5 years (20.4 vs 16.2 mg/day, *P* < 0.001) and 7 years (20.2 vs 16.7 mg/day, *P* = 0.002) of treatment.

Supplementary Figure 1 shows the probability of non-control of disease over time, according to the combination of the initial type of medical therapy for acromegaly and the basal IGF-1 value, i.e. the two relevant factors that emerged from multivariate analyses. Patients treated with pegvisomant monotherapy and having baseline IGF-1 values <300 (dark green line) or 300–500 µg/L (orange line) are those with the lowest probabilities of uncontrolled acromegaly at most time points of the first 10 years of follow-up, with the latter combination showing the steepest decrease in the probability over the years.

Supplementary Figure 2 shows the receiver operating characteristic (ROC) curve from multivariate analyses, including terms for medications for acromegaly and IGF-1 value at baseline. The area under the curve (AUC) was 0.72, indicating that an acceptable predictive model was fitted.

## Discussion

In this study, we explored factors that could influence and predict the effectiveness of pegvisomant therapy to control acromegaly. Starting from the whole cohort of ACROSTUDY patients, we selected 1546 subjects for which adequate data are available. We then performed longitudinal, multivariate analyses considering a large number of covariates potentially related to disease control prediction. Our results confirmed and for the first time quantified the central role of the baseline IGF-1 values on the probability of achieving a biochemical control of acromegaly during the treatment with pegvisomant. Patients with a baseline IGF-1 value below 500 µg/L had a lower risk of having an uncontrolled acromegaly over time, while those with IGF-1 ≥700 µg/L showed a higher risk. A potential role of the treatment strategy also emerged, with pegvisomant monotherapy reporting the lowest risk of missing the control of acromegaly over the years, compared to different pegvisomant-based combination therapies.

Noteworthy, no differences of pegvisomant doses were observed between patients with controlled disease and those with un-controlled acromegaly. The main reason to explain this lack of differences could be a certain therapeutic inertia, associated with both a possible failure of health care providers to initiate or intensify therapy when indicated and poor patient adherence. On the one hand, pegvisomant may be underdosed and a disease control may be not achieved. Indeed, in another investigation on ACROSTUDY, high doses of pegvisomant (above the allowed maximal dose of 30 mg daily) normalized IGF-1 in younger patients, with more aggressive disease, more hypertension, sleep apnea, diabetes, and overweight and with higher baseline levels of IGF-1 compared to patients who were treated with lower doses of pegvisomant (26). Furthermore, it should be considered that even difficulties with insurance coverage and medication access may be barriers to dose titration. On the other hand, data from the German Acromegaly Registry Database showed that patients who reported long-standing active acromegaly were less motivated to agree to therapeutic recommendations, and noncompliance with medical therapy was observed (27). Interestingly, when patients were included in a controlled trial, they were subjected to periodic follow-up and their treatment plan recorded any missed doses, ruling out, with high probability, there was no poor treatment adherence. The possible role of the treating physician in the failure to titrate pegvisomant and the inadequate adherence should be addressed in the future to optimize pegvisomant effectiveness in real world.

Earlier studies examined the predictive effect of patient- or disease-related characteristics on the response to the treatment and to the control of acromegaly. Several of them showed a detrimental role of high baseline IGF-1 values. A previous investigation based on the German cohort of ACROSTUDY found that higher IGF-1 values and higher BMI at baseline were inversely associated with the normalization of IGF-1 values after 1 year of pegvisomant therapy (17). Some studies reported that higher doses of pegvisomant were required to normalize serum IGF-1 levels in patients with high IGF-1 at baseline (18, 20, 28, 29). In our analysis, pegvisomant dose and baseline IGF-1 were strictly connected. Pegvisomant dose over time was higher in uncontrolled than in controlled patients. It was thus inversely associated with disease control in univariate analyses (likely for a reverse causation effect, i.e. increasing doses were given to patients with severe, difficult-to-control disease). Still, the association vanished after adjusting for IGF-1 at baseline in multivariate analyses. Thus, pegvisomant dose did not appear to play a significant (neither favorable nor detrimental) effect on disease prognosis after correcting for acromegaly severity. Two other studies from South America also supported a key role of pretreatment IGF-1 levels on response to pegvisomant (either in monotherapy or combined therapy), in the absence of other predictors of response identified in both analyses (10, 30). Thus, our findings are consistent with earlier results and provide further evidence and quantification of the issue using extensive prospective information and robust statistical methods.

Previous studies reported an effect of age, sex, body weight or BMI, and prior radiotherapy, on acromegaly control, showing inconsistent results (17, 18, 19). In univariate analyses, we observed associations suggestive of a lack of disease control in patients initiating pegvisomant treatment at a younger age (i.e. <40 years) and in obese patients. Similarly, in another observational study, the lack of acromegaly control was associated with higher BMI at baseline and was more frequent with BMI >30 kg/m^2^ (20). Although in ACROSTUDY French Registry, mean body weight and BMI increased from baseline to 5 years’ follow-up (31), in this study we collected information on BMI only at baseline. At the time of the Fleseriu publication in 2021 (24), we looked at BMI longitudinally and did not see a significant effect of increased BMI over the years. Concerning the effect of radiotherapy, it was not significant in the univariate screening process (*P* = 0.1514); the probability of non-IGF control was 0.41 in patients undergone radiotherapy compared to 0.40 in the cohort of patients who did not receive radiotherapy. No relation emerged after accounting for other covariates in the multivariate analysis; therefore, our findings did not support a differential impact of these factors on the outcome of pegvisomant treatment.

We found a lower probability of not controlling the disease in patients treated with pegvisomant monotherapy compared to patients treated with a combination regimen, even after adjusting for the IGF-1 at baseline and for pegvisomant dose. This finding should be interpreted with caution since no such results were reported in previous studies. However, in a previous real-life study (32), it was observed that the number of patients controlled under pegvisomant monotherapy was significantly higher than patients treated by a combined therapy (pegvisomant/SSA). It should be noted that in the same study, patients who needed to be treated with combined therapy had higher IGF-1 levels at baseline than those treated with monotherapy. Also, a potential role of residual confounding factors (particularly related to the severity of disease) cannot be excluded. Furthermore, a significant interaction between the time and the treatment with pegvisomant emerged, thus indicating that the effect of different treatments (i.e. monotherapy and combination therapies) on the probability of not controlling acromegaly changed over time. Moreover, our data show that IFG-I levels at baseline are similar both in patients treated with Peg alone and a combination regimen (Supplementary Table 1). On the other hand, the latter treatment seems to be related to a worse response compared to Peg alone, according to either our experience and previous ACROSTUDY experiences (27, 32). Several reasons might explain what we observe: the lower Peg dose administrated in combination therapy compared to the one in Peg alone, the differences in body weight, as shown in Tables 1 and 2, or the barriers to dose titration, such as physician inertia in adjusting the dose, insurance coverage and medication access itself, which could occur more commonly during the combination treatment than in a single-therapy approach.

This study, conducted in a real-world setting, has several limitations and strengths. Potential bias and information limits are inherent to the observational design of ACROSTUDY and of the present analysis. Bias may originate from the selective inclusion in the study of severe acromegaly patients (e.g. pretreated ones who had experienced treatment failures) or the selective drop-out of patients non-responding to treatment or experiencing unsatisfactory outcomes during the follow-up. As regards information limits, the study was focused mainly on personal and clinical characteristics, and no information on biomarkers or genetics could be included in the regression models; in real-world studies, collection of data on treatment (e.g. dose at a given time point) is less accurate than in RCT, and it is also difficult to ensure appropriate titration/dosing. Furthermore, as discussed earlier, we cannot exclude the presence of some residual confounding in multivariate analyses. We tried to overcome at least part of these limits through appropriate statistical modeling: by using mixed-effects models, allowing us to take into account changing values of covariates at yearly visits and to prevent listwise deletion due to missing data, and by adopting multivariate analyses, adjusted for several covariates. The multivariate model also showed satisfactory goodness of fit (Supplementary Fig. 2). Besides limitations, observational investigations like ACROSTUDY have important strengths. They provide valuable findings from real-life clinical practice in unselected patient populations. Other strengths of this analysis are its longitudinal design, international study setting and large sample size.

## Conclusion

In conclusion, we have provided new evidence on the impact of several factors on acromegaly disease control among patients treated with pegvisomant from a large, prospective study including data from 15 countries. Although it is difficult to quantify the role of therapeutic inertia, our findings strongly highlight the key role of IGF-1 level at baseline and do not support an effect of age, sex, BMI and pegvisomant dose on the probability of achieving disease control.

## Supplementary materials

Supplementary Table 1. Mean ± SD of IGF-1 value and of pegvisomant daily dose at different treatment times in two patient subgroups treated during the whole period of the first 24 months with: i) pegvisomant only; and ii) with pegvisomant plus SSA, according to disease control at different time points.

Supplementary Table 2. Mean ± SD of pegvisomant daily dose at different treatment times in patients with controlled and non-controlled acromegaly.

Supplementary Figure 1. Probability of uncontrolled IGF-1 values over time, according to the combination of factors significant in multivariate analyses (i.e., medications for acromegaly and IGF-1 value at baseline).

Supplementary Figure 2. Receiver Operating Characteristic (ROC) curve from multivariate longitudinal mixed-effect logistic regression model including terms for medications for acromegaly and IGF-1 value at baseline.

Supplementary Material

## Declaration of interest

Martin Ove Carlsson, Roy Gomez and Simona Granato are employees and stockholders of Pfizer. Silvia Grottoli received research grants from Pfizer. Salvatore Cannavò received fees from Pfizer during the last 2 years for participation to scientific boards. Other authors have no conflicts of interest to declare.

## Funding

ACROSTUDY was designed and sponsored by Pfizerhttp://dx.doi.org/10.13039/100004319. Editorial support was provided by Content Ed Net, with the helpful assistance of Claudio Pelucchi and Carlotta Galeone, and was funded by Pfizerhttp://dx.doi.org/10.13039/100004319.
