# Supplementary material for: Factors associated with disease control failure in acromegaly patients treated with pegvisomant: an ACROSTUDY analysis
Source: Endocr Connect. 2024 Jan 29;13(3):e230247. doi: 10.1530/EC-23-0247 (PMC10895310; doi:10.1530/EC-23-0247)
Supplement: Supplementary Table 1. Mean ± SD of IGF-1 value and of pegvisomant daily dose at different treatment times in two patient subgroups treated during the whole period of the first 24 months with: i) pegvisomant only; and ii) with pegvisomant plus SSA, according to disease control at different time poin [file supplementary_table_1.pdf]

Article title: FACTORS ASSOCIATED WITH DISEASE CONTROL FAILURE IN ACROMEGALY PATIENTS: AN ACROSTUDY ANALYSIS

Journal name: Pituitary

Author names: Antonella Giampietro, Sabrina Chiloiro, Claudio Urbani, Rosario Pivonello, Martin Ove Carlsson, Francesca Dassie, Nunzia Prencipe, Marta Ragonese, Roy Gomez, Simona Granato, Salvatore Cannavò, Silvia Grottoli, Pietro Maffei, Annamaria Colao, Fausto Bogazzi, Antonio Bianchi.

Corresponding Author: Antonella Giampietro, Pituitary Unit, Fondazione Policlinico Universitario A. Gemelli IRCCS, antonella.giampietro@policlinicogemelli.it

Supplementary Table 1. Mean ± SD of IGF-1 value and of pegvisomant daily dose at different treatment times in two patient subgroups treated during the whole period of the first 24 months with: i) pegvisomant only; and ii) with pegvisomant plus SSA, according to disease control at different time points.

**Treatment timing / measure**	**Patients treated with peg only (n=860) (Mean ± SD)**	**Patients treated with peg/SSA (n=491) (Mean ± SD)**
**Baseline**		
IGF-1 value (ug/L)	500.1 ± 275.8	461.5 ± 330.3
Pegvisomant dose (mg/day)	12.3 ± 7.9	9.3 ± 5.8

Treatment timing / measure	Patients treated with peg only (n=860) (Mean ± SD)				Patients treated with peg/SSA (n=491) (Mean ± SD)			
**After 12 months**	**NCD12 (n=305)**		**CD12 (n=197)**		**NCD12 (n=140)**		**CD12 (n=130)**	
Pegvisomant dose (mg/day)	15.0 ± 7.0		16.2 ± 6.9		12.3 ± 6.6		12.8 ± 7.9	
**After 24 months**	**NCD12-NCD24 (n=85)**	**NCD12-CD24 (n=68)**	**CD12-NCD24 (n=61)**	**CD12-CD24 (n=181)**	**NCD12-NCD24 (n=58)**	**NCD12-CD24 (n=49)**	**CD12-NCD24 (n=28)**	**CD12-CD24 (n=77)**
Pegvisomant dose (mg/day)	17.7 ± 7.5	17.6 ± 7.7	16.7 ± 9.4	15.1 ± 6.9	14.1 ± 8.5	14.2 ± 7.4	13.9 ± 9.1	12.1 ± 5.7

NCD12: not controlled disease at 12 months; CD12: controlled disease at 12 months; NCD24: not controlled disease at 24 months; CD24: controlled disease at 24 months
