# Supplementary material for: Factors associated with disease control failure in acromegaly patients treated with pegvisomant: an ACROSTUDY analysis
Source: Endocr Connect. 2024 Jan 29;13(3):e230247. doi: 10.1530/EC-23-0247 (PMC10895310; doi:10.1530/EC-23-0247)
Supplement: Supplementary Table 2. Mean ± SD of pegvisomant daily dose at different treatment times in patients with controlled and non-controlled acromegaly. [file supplementary_table_2.pdf]

Article title: FACTORS ASSOCIATED WITH DISEASE CONTROL FAILURE IN ACROMEGALY PATIENTS: AN ACROSTUDY ANALYSIS

Journal name: Pituitary

Author names: Antonella Giampietro, Sabrina Chiloiro, Claudio Urbani, Rosario Pivonello, Martin Ove Carlsson, Francesca Dassie, Nunzia Prencipe, Marta Ragonese, Roy Gomez, Simona Granato, Salvatore Cannavò, Silvia Grottoli, Pietro Maffei, Annamaria Colao, Fausto Bogazzi, Antonio Bianchi.

Corresponding Author: Antonella Giampietro, Pituitary Unit, Fondazione Policlinico Universitario A. Gemelli IRCCS, antonella.giampietro@policlinicogemelli.it

**Supplementary Table 2.** Mean ± SD of pegvisomant daily dose at different treatment times in patients with controlled and non-controlled acromegaly.

| | Pegvisomant mean ± SD daily dose | | |
|---|---|---|---|
| Treatment timing | Patients not controlled | Patients with controlled disease | p-value |
| 1 year | 14.6±8.2 | 14.4±7.3 | 0.5839 |
| 3 years | 18.1±10.4 | 15.0±7.8 | <0.0001 |
| 5 years | 20.4±12.3 | 16.2±8.8 | <0.0001 |
| 7 years | 20.2±11.7 | 16.7±9.8 | 0.0024 |
| 10 years | 20.3±10.5 | 17.7±9.7 | 0.1523 |
