# Supplementary material for: Factors associated with disease control failure in acromegaly patients treated with pegvisomant: an ACROSTUDY analysis
Source: Endocr Connect. 2024 Jan 29;13(3):e230247. doi: 10.1530/EC-23-0247 (PMC10895310; doi:10.1530/EC-23-0247)
Supplement: Supplementary Figure 1. Probability of uncontrolled IGF-1 values over time, according to the combination of factors significant in multivariate analyses (i.e., medications for acromegaly and IGF-1 value at baseline). [file supplementary_figure_1.pdf]

Article title: FACTORS ASSOCIATED WITH DISEASE CONTROL FAILURE IN ACROMEGALY PATIENTS: AN ACROSTUDY ANALYSIS

Journal name: Pituitary

Author names: Antonella Giampietro, Sabrina Chiloiro, Claudio Urbani, Rosario Pivonello, Martin Ove Carlsson, Francesca Dassie, Nunzia Prencipe, Marta Ragonese, Roy Gomez, Simona Granato, Salvatore Cannavò, Silvia Grottoli, Pietro Maffei, Annamaria Colao, Fausto Bogazzi, Antonio Bianchi.

Corresponding Author: Antonella Giampietro, Pituitary Unit, Fondazione Policlinico Universitario A. Gemelli IRCCS, antonella.giampietro@policlinicogemelli.it

Supplementary Figure 1. Probability of uncontrolled IGF-1 values over time, according to the combination of factors significant in multivariate analyses (i.e., medications for acromegaly and IGF-1 value at baseline).

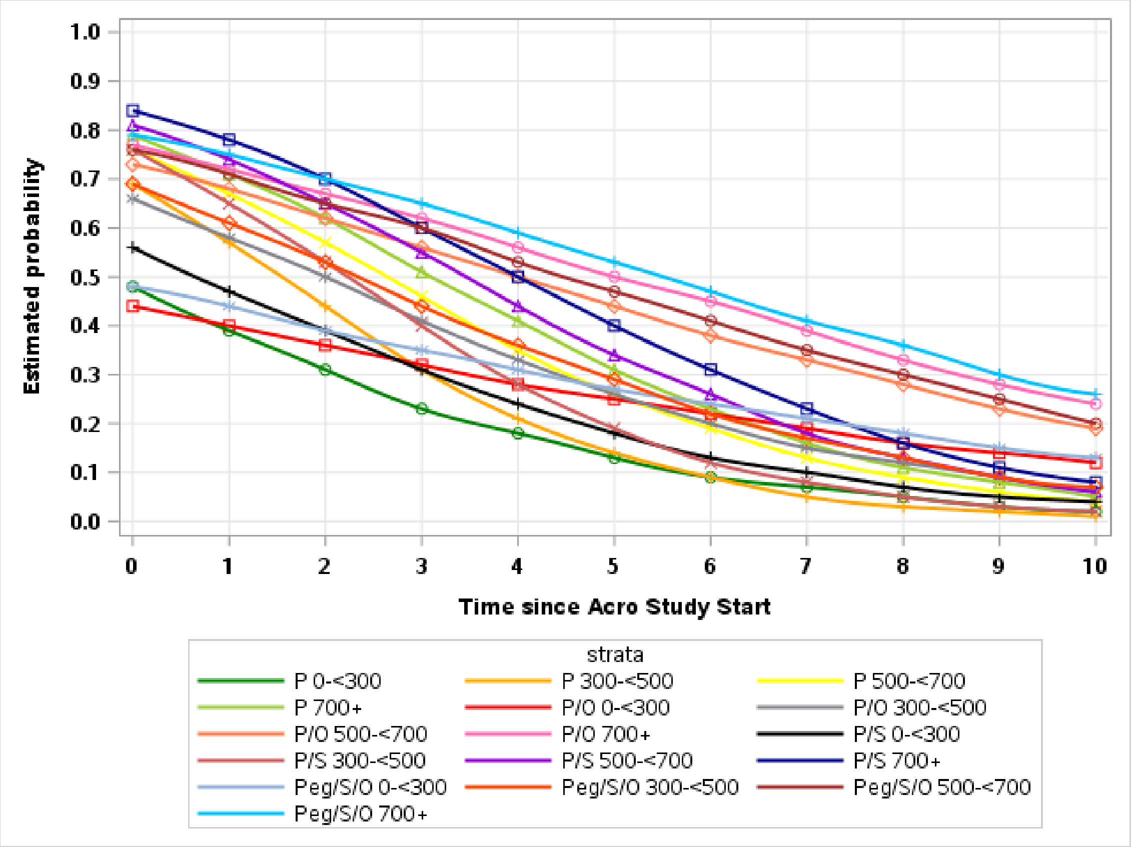

P: pegvisomant only; P/O: pegvisomant and other (non-SSA); P/S: pegvisomant and SSA; Peg/S/O: pegvisomant, SSA and other; 0-<300, 300-<500, 500-<700 and 700+ are subgroups of IGF-1 value at baseline (ug/L)
