# Supplementary material for: Factors associated with disease control failure in acromegaly patients treated with pegvisomant: an ACROSTUDY analysis
Source: Endocr Connect. 2024 Jan 29;13(3):e230247. doi: 10.1530/EC-23-0247 (PMC10895310; doi:10.1530/EC-23-0247)
Supplement: Supplementary Material [file supplementary_material.pdf]

Supplementary material.

The following drugs were used in ACROSTUDY.

Lanreotide SR, Lanreotide Autoqel, Octreotide LAR, Octreotide short acting, Pasireotide LAR, Pasireotide (SOM 230 LAR) , SOM 230, Somatostain, Sandostatin, Sandostatin LAR 30mg), Bromocriptine, Caberqoline, Quinaqolide, Other(Lisuride, Terqurid, Terquride, Dostinex, Noprolac, Parlodel), Pasioetid, Pasireotid LAR, Siqnifor, Sandostatin LAR, Somatostain, Somatostain analog – long acting, Somatuline depot, Somatuline depot/Lanreotide , Somavert, Ocreotide LAR, octreotide and ITF2984, Somatuline depot.

These drugs, along the manuscript, are referred collectively as “other”.

The following information were collected in the ACROSTUDY [Brue et al. 2009]:

- Date of informed consent.
- Time of first diagnosis of acromegaly.
- Physical examination (including height, weight, blood pressure) including acromegaly related co-morbidities.
- Previous and current therapy for acromegaly; concomitant medication.
- IGF1 levels at baseline, i.e. at commencement of pegvisomant, after 6 months of pegvisomant treatment and every 6 months thereafter
- Pituitary function and hormone replacement therapy.
- Pituitary imaging studies (i.e. magnetic resonance imaging (MRI), CT scan) at baseline (defined as pegvisomant start), 6 and 12 months post pegvisomant treatment start and then annually.
- Visual fields.
- Liver function tests (ALT, AST), fasting blood glucose and HbA1c (diabetic patients only)
- Symptoms (patient-assessed acromegaly symptom questionnaire, PASQ) of acromegaly during the study.
- Adverse events.
